# Postabortal Group A Streptococcal Sepsis and Toxic Shock Syndrome

**DOI:** 10.1155/S1064744997000331

**Published:** 1997

**Authors:** David E. Soper

**Affiliations:** Department of Obstetrics and Gynecology Medical University of South Carolina Charleston SC 29401 USA


					Infectious Diseases in Obstetrics and Gynecology 5:210 (1997)
(C) 1997 Wiley-Liss, Inc.
Images in Infectious Diseases in Obstetrics and Gynecology
Section Editor: David E. Soper, M.D.
Postabortal Group A
Streptococcal Sepsis and
Toxic Shock Syndrome
David E. Soper, MD
Department of Obstetrics and Gynecology, Medical University
ofSouth Carolina, Charleston, SC 29401
LEGEND TO FIGURE
A 20-year-old woman developed lower abdominal pain
and fever within 48 h of undergoing an elective termi-
nation of a 10 week pregnancy by suction dilation and
curettage. Progressive clinical deterioration prompted an
exploratory laparotomy which revealed necrotic ovaries
bilaterally (A). Areas of the myometrium were also ne-
crotic and hemorrhagic (B). An abdominal hysterectomy
and bilateral salpingo-oophorectomy was performed.
(C) 1997 Wiley-Liss, Inc.
Images in Infectious Diseases in Obstetrics and Gynecology presents clinically important visual images that a practitioner in women's health
might encounter. If you have a high-quality color or black-and-white photograph of slide representing such an image that you would
like considered for publication, send it with a descriptive legend to David E. Soper, MD, Medical University of South Carolina, 171
Ashley Avenue, Charleston, SC 29401-2233.
Images is made possible through an educational grant from Pfizer, Inc.

				

